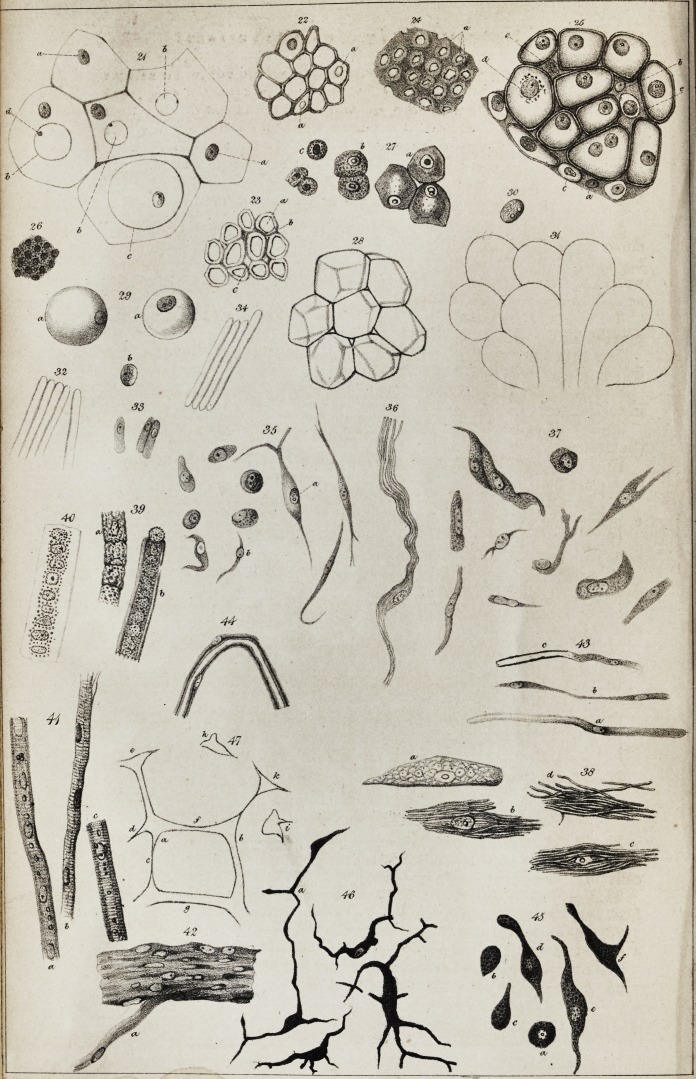# Microscopical Researches Respecting the Identity of Structure and Mode of Growth in Animals and Plants

**Published:** 1840-04

**Authors:** 


### ART. X.

*Mikroskopische Untersuchungen über die Uebereinstimmung in der Struktur und dem Wachsthum der Thiere und Pflanzen.* Von Dr. TH. SCHWANN. Mit 4 Kupfertafeln.—*Berlin*, 1839. 8vo, pp. 270.

*Microscopical Researches respecting the Identity of Structure and mode of Growth in Animals and Plants.* By Dr. THOMAS SCHWANN.—*Berlin*, 1839.

THE ardour and success with which the study of anatomy and physiology has been prosecuted at Berlin during the last few years are well known. Of the zealous enquirers who have contributed thus to raise the character of the university of the Prussian capital in the departments of science which we have mentioned, the author of the work before us is one of the most industrious, and, judging from the quality of his labours, one of the most ingenious and scientific.

M. Schwann has already gained a reputation in England by his important observations on digestion, published in Müller's Archiv for 1836; but as early as 1834 he had distinguished himself by the performance of well-directed experiments, which seemed to demonstrate in a conclusive manner the necessity of the access of atmospheric air for the development of the embryo of the bird.* Since that time, he has made known the results of several investigations into the microscopic structure of the muscular and other tissues, and very recently published some highly ingenious experiments,† which go far to establish the fallacy of the hypothesis of equivocal generation as hitherto maintained. The work before us is, however, of a very different character from any of those productions, since its object is not to demonstrate an isolated fact, or to determine the accuracy or inaccuracy of a doubtful hypothesis, but to establish the existence of a hitherto unknown but important law, of general application to all organized beings.

"The object of the present treatise," says M. Schwann in his preface, "is to demonstrate the intimate relation in which the two kingdoms of organic nature stand to each other, by showing the identity of the laws of development of the elementary parts of animals and plants. The main result of the enquiry is, that the law of development of the elementary parts of all organisms is the same, just as all crystals, however different their forms, are developed in accordance with the same laws." (p. 4.)

Analogies between the elementary composition of animals and that of plants have been frequently pointed out; but the more recent advances in minute anatomy have tended to prove that no real similarity exists between the elementary parts of these two great classes of organized bodies. Thus, it had been said that cellular tissue formed the groundwork of animal as of vegetable textures; but, on more accurate investigation, it appeared that the cellular tissue of animals was a very different structure from that of plants,—that while the latter was composed of vesicles or cells closed on all sides, the former consisted essentially of fibres aggregated into bundles and laminæ, and interwoven so as to

* De necessitate aeris atmosphærici ad evolutionem pulli in ovo incubito. Dissertatio Inauguralis, 1834. Berol. iv.

† Poggendorf's Annal., 1837. Bd. xli., p. 184.

inclose cavities which communicated freely with each other. Again, it had been asserted that the great bulk of animal tissues was made up of the same elements as those constituting the whole vegetable organism, namely, membrane and fibre; but the microscope has shown that the most abundant fibres of plants, the woody fibres, are really membranous tubes, and on the other hand that the membranes of animals which were compared to the simple vegetable membranes, are compound structures formed by the interlacement of fibres. More recently it has been affirmed that the ultimate elements of all the solid textures of animals, as well as of plants, are globules,—that the fibres of animal cellular tissue, for example, are mere strings of globules; but the appearances on which this statement was founded are now known to be in many instances illusory, and dependent on the imperfection of the microscope employed, or on inattention to certain necessary precautions in its use.

In fact, in proportion as the microscope has been improved, and the knowledge obtained by its means consequently rendered more accurate, the positive characters of the elementary tissues of animals and plants have appeared more and more dissimilar. The simplicity of vegetable structures, as M. Schwann remarks, has become more manifest. The plants of one entire class have received their designation of "cellulares," from the circumstance of their being entirely formed of cells. And although the perfectly-developed "vascular" plants contain other elements, namely, woody fibre, vessels, and ducts, yet these are now known to be cells altered in form, either by simple elongation or by the coalition of several to constitute one tube. In animals, on the contrary, a more intimate acquaintance with their minute structure has seemed only to reveal a greater and greater multiplicity of component elements, perfectly distinct from each other, and still more unlike the closed cells of vegetables. It is true that, before M. Schwann commenced his researches, some animal tissues had been observed to resemble, very accurately, the cells of vegetable parenchyma in form. Thus, the similarity of the closed cells composing the adipose substance to the vegetable cells, could not escape notice; some forms of epithelium and of cartilage (that of the branchiæ of the tadpole) had been compared by Purkinje and Valentin* to vegetable cellular tissue; and the perfectly plant-like form of the cells of the chorda dorsalis of young embryos and fishes had been pointed out by Müller.† These facts, however, appeared of little importance, and no general principle was deduced from them. It was not, indeed, in their perfect forms but in their mode of development that the great points of resemblance between the tissues of plants and those of animals were to be found. This was perceived by M. Schwann, and suggested to his mind the idea of comparing the growth of animal and vegetable cells, with the view of demonstrating a general law of development for all organic elementary tissues.

"When I entered upon my enquiry," he remarks, "there appeared to be no unity in the mode in which the organic molecules were aggregated together to form the different tissues. Here they met to form a fibre, there to form a cell, and here again to constitute a globule. The principle of development appeared to be totally different for all elementary parts of which the physiological en-

* Entwickelungs-geschichte, pp. 209-10.

† Anatomie der Myxinoiden, p. 74.

dowments were not the same. And while the laws regulating the development of a cell and a fibre were admitted to be different, it was necessarily supposed that those to which the development of the different kinds of cells or of the different kinds of fibres was subject, were also different, though not in the same degree." (p. 12.)

The observations of M. Schleiden,* however, on the development of vegetable cells, revealed the existence of certain definite steps in the process, and afforded to Schwann adequate data for comparing the mode of growth of cells in the animal and vegetable organisms. He was thus led to the discovery, "that all the manifold forms of animal tissue are produced by the transformation of cells, wholly analogous to those of plants in structure, and for the most part agreeing with them most remarkably in the phenomena of independent vegetative life, which they present." (p. 2.)

Before proceeding to the consideration of M. Schwann's observations on the different animal tissues, we shall lay before our readers some account of those of M. Schleiden, on which they are based, and to their accordance with which they owe much of their interest and importance.

All newly-forming tissues of plants appear to take their origin from *gum*. This substance is in some instances directly supplied by the operation of the formative processes upon the nutritive materials derived from without. More frequently, however, it has passed through the intermediate condition of *fecula* (starch), or one analogous to it. Starch, in the plant, appears to take the place of animal fat. It is superfluous nutritive matter, deposited for future use; and it is found most abundantly in those parts of the structure in which, after a short repose, a new formative process is to commence; whilst it is absent in those which are in a state of active growth. Much diversity has existed between the accounts recently given of the nature of this substance. According to Raspail, it consists of membranous vesicles containing a gummy fluid; these vesicles being formed within the cells of the plant, and taking their origin from them. The later researches of M. Payen, however, leave but little room for doubt that the supposed membrane cannot be regarded as having an organic structure; but that it is rather the concrete exterior of the gummy matter deposited in the cell, often thickened by a coating of other substances. The increase of the granule takes place by absorption into its interior; so that there is a series of concentric layers, in each granule of fecula, gradually approaching the fluid character as we advance from without inwards. Starch only presents this definite aspect, however, when slowly deposited in the internal parts of plants, where it is to remain unchanged for some time. In cells more actively concerned in the formative processes, such as those of the leaf, the pollen, and the albumen of the seed, we find it existing in smaller granules, which float in the fluid contents of the cell. The form of these granules is extremely various; but they are distinguished by the colour they take when brought into contact with iodine. Simple and unimportant as the fact appeared that iodine produces this change, it has had a most important influence on the subsequent progress of

* Müller's Archiv, 1838, p. 137.

vegetable physiology, by enabling the microscopic observer to ascertain the real character of particles of extreme minuteness, whose form alone would have left him in doubt.

Under whichever of these conditions fecula exists in the plant, it is converted into gum before it can be concerned in the production of new tissues. This conversion appears to be a purely chemical change; and it may be brought about in the laboratory under conditions similar to those afforded by the vegetable organism. In the state which immediately precedes the commencement of organization, gum appears as a consistent fluid, slightly wanting in transparency; and it is coagulated granularly by the tincture of iodine, with a pale yellow permanent colour. In the further process of organization, a quantity of extremely minute granules appear in the gummy fluid; most of which, on account of their minuteness, are seen merely as black points. The fluid then seems to take from iodine a somewhat darker yellow; but the granules, when their size enables their colour to be distinguished, seem to become by this process of a dark brownish yellow.

It is probable that, in all plants, the chemical state of the materials which form cells is nearly the same; but there is not the same uniformity in the mode in which cells are generated. The researches of Schleiden refer almost entirely to the phanerogamia; and, as we shall hereafter see, they are not to be regarded as even generally applicable to the lower tribes of plants, without much further enquiry. We are not prepared to admit that the formation of new cells in flowering plants always takes place in this manner; since the observations of Schleiden have been as yet chiefly confined to parts in which we can easily imagine some peculiarities to exist. He specifies the large cell or embryonal sac of the unimpregnated ovule (in which the albumen is afterwards formed), and the end of the pollen-tube (from which the cells of the embryo itself are developed), as the points in which the process of organization may be observed most easily and clearly.

At both these places, the fluid is at first homogeneous and transparent; but the minute granules before mentioned soon originate in it; and it then becomes opalescent, or, through the presence of a larger mass of granules, even opaque. Single, larger, more sharply defined granules are now evident in this mass (fig. 1, plate ii.); and very soon afterwards these present a regular form, and increase considerably in size, apparently from the coagulation of the minuter granules around the larger ones. From these bodies the cells take their origin in the mode to be presently described; and hence Schleiden proposes for them the name of *cytoblast* (κυτος, cell, βλαστος, germ). These bodies had been previously observed in the newly-formed cells of some plants, and in the adult tissue of a few others, by Robert Brown and other observers; but none had enquired into their relation with the original construction of the cell. Their form varies from oval to circular; sometimes they are flattened, or lenticular; sometimes almost spherical. Their colour is in general yellowish, yet sometimes passing almost into a silver white, and occasionally almost transparent. The internal structure of the cytoblast is mostly granular; without, however, the granules of which it consists being clearly distinct from one another. Its consistence is very various; from such a softness that it almost dissolves into water, to that degree of firmness that it bears even the pressure of the compressorium without losing its form. The nearer it is to its origin, the softer it is; and it has less consistence where it ultimately disappears, as it usually does, than where it remains throughout the life of the plant.

Even the cytoblast, however, does not appear to be the real elementary organ. On the larger and best developed forms of these there is observed a small sharply-defined body, which seems to have the form of a thick ring, or a thick-walled hollow globule (fig. 2). In less developed cytoblasts, only the outer sharply-defined circle of this ring can be observed; and in its centre a dark point (fig. 3). In still smaller cytoblasts it appears merely as a sharply circumscribed spot; or, lastly, there is observed only a remarkably small dark point. In the very smallest and most transitory cytoblasts (for instance in the leaves of dicotyledons), Schleiden has not hitherto been able to discover it. In a few rare cases, two or even three of these nucleoli have been seen in one cytoblast (fig. 3). From his observations on all plants which admitted of a complete watching of the entire process of formation, Schleiden concludes that these small bodies are formed earlier than the cytoblast; and he suggests that they may have some relation with the nucleoli of the starch-granules. The size of this corpuscle varies considerably, from the extent of half the diameter of the cytoblast to the most minute point whose size did not allow of measurement. Sometimes it appears darker and sometimes brighter than the remaining mass of the cytoblast. In general, it has more consistency than the latter, and still continues sharply defined when this has been changed by pressure into an amorphous mucus.

When the cytoblasts, increasing separately in the gummy fluid, have attained their full size, the formation of the cells commences. A delicate transparent vesicle is seen on the surface of each, in the form of a flat segment of a sphere, whose plane side is composed of the cytoblast, and the convex side of the membrane of the young cell, which is situated on the cytoblast somewhat like a watch-glass on a watch. The space between the two is perfectly clear, and appears filled with aqueous fluid Externally the membrane is in contact with the mucous granules of the surrounding fluid, which are pressed back by its expansion. The appearance at this period is represented in fig. 4; and, on a smaller scale, in fig. 5, *a*. If the vesicles are isolated, the mucous granules may be almost entirely removed by shaking the stage; but the vesicles themselves also soon disappear, dissolving entirely in distilled water, and leaving only the cytoblasts behind. As the process of formation advances, the vesicle gradually extends, and becomes more consistent (fig. 5, *b*); it increases beyond the margin of the cytoblast, and quickly becomes so large, that at last the latter merely appears like a small body inclosed in one of its side walls. At the same time the young cell frequently exhibits irregular indentations (fig. 5, *c*); a proof that the expansion by no means proceeds uniformly from one point. After further growth of the cells, their form becomes more regular; and when their sides begin to press against one another, they are flattened. If the pressure be equal in all directions, the form assumed by the cell will be that of a rhomboidal dodecahedron; and this is often observed in the pith and other parts in which the cellular tissue is not too much compressed. The cytoblast is still inclosed in the wall of the cell; but at a later period it is usually absorbed, especially when the cell is destined to undergo still further development. In the *orchideæ* and *cacteæ*, a considerable part of the cellular tissue exhibits the cytoblasts during the whole of life. It was in the former that this body was first observed by Brown, who denominated the opaque spot which he there perceived the *areola* or nucleus. It remains persistent in many hairs, especially those which are articulated, and exhibit circulation of fluid in their cells. It is a curious fact, which proves the relation between the presence of the cytoblast and the vital activity of the cell, that the currents which frequently cover the whole wall in a network of streams, always proceed from it and return to it. In fig. 6 is seen a portion of the hair of a potato, as represented by Schleiden, with the persistent cytoblasts and currents. And in fig. 7 is shown the course of the currents in one cell of the beaded hair of the tradescantia virginica, as observed some years since by Brown, and subsequently by Slack.* Sometimes, on the contrary, not only the cytoblast is reabsorbed, but the cell itself. This it is not difficult to conceive, when it is known that, up to the period when the sides of the adjacent cells become flattened against each other, the want of consistency in their walls is such that the membrane may be easily caused to disappear, or, as it were, to dissolve away, by agitation. Reabsorption never takes place at a more advanced period of development, when the original membrane has not only become more perfectly organized, but has been strengthened by secondary deposits within its cavity, in the manner to be hereafter described. It would seem that the membrane of the fully-formed cell consists of two laminæ, between which the cytoblast is inclosed (fig. 8). The inner one is the most delicate, and frequently only gelatinous; and this is reabsorbed at the same time with the cytoblast.

In connexion with these observations, we shall now give a brief analysis of the researches of Schleiden on the generative process in the phanerogamia. It will be seen that, according to his view, the early condition of the vegetable embryo precisely resembles the permanent state of the simplest cellular plants; and that its production takes place in accordance with the principles which have just been expounded. In a former article on the state of vegetable physiology (Vol. IV., p. 29), we mentioned the well-ascertained fact that prolongations of the membrane of the pollen-grains which fall upon the stigma find their way down the style, and apply themselves to the foramen of the ovule; and we stated it is probable that, from the contents of the pollen-grain thus conveyed into the ovule, the embryo, which subsequently appears, takes its origin. The researches of Schleiden have confirmed this view, and have given it a much greater degree of precision. According to his statements, the cavity of the ovule before impregnation is occupied by a large vesicle containing a turbid fluid ready to become organized in the mode already mentioned. The pollen-tube which enters the cavity, through the foramen left by the imperfect closure of the coats of the seed, pushes the membrane before it (fig. 9); and it thus becomes gradually imbedded in the embryo-sac, as, according to the Hunterian doctrine, the ovum acquires a double coating from the membrana deci-

* Transactions of the Society of Arts, vol. xlix.

dua. By absorption through the membranes, the nutritious materials contained in the embryo-sac appear to find their way into the pollen-tube; and they thus contribute to the development of the cell-germs contained in the latter. The starch originally contained in the pollen-grain usually changes its character in its descent along the tube; so that the fluid at the embryonal extremity of the latter is transparent and colourless. Here the development of cells takes place in the mode already described; and it is often observed to occur not only in that part of the pollen-tube contained within the ovule, but in the free portion also. The appearance presented by the pollen-tube of *orchis morio* is shown in fig. 10, where the cells are seen to be formed from cytoblasts within the general tubular envelope. In the part of the tube which is imbedded in the ovule, the development of the cells does not go on in a single row merely; but the tube expands in such a manner as to contain a cluster, as is shown in fig. 11. Schleiden states that, even after the development of cells within the pollen-tube had commenced, he was able to withdraw the tube from its bed in the embryo-sac, especially in orchideous plants. After a time, however, the newly-forming cells increase so much, that the enveloping membrane of the pollen-tube is no longer recognizable. Within the cells first produced many new ones are generated, and the membranes of the parent cells disappear in their turn. The embryo is nourished by absorption through the embryonal sac. In the higher dicotyledonous plants, its increase continues until it occupies the whole seed, having entirely exhausted the contents of the embryo-sac. In monocotyledons and some dicotyledons, however, the development of the embryo does not advance so far before the maturation of the seed; and the contents of the embryo-sac, not being absorbed into the embryo, develope themselves into cells externally to it, forming what is known to botanists as the *albumen* of the seed. It is in this situation that the process of the formation of cells has been chiefly observed by Schleiden.

We shall now proceed to enquire into the principal changes which the cells, from which all vegetable structures take their origin, subsequently undergo.

It has been justly remarked by Schleiden, that every cell leads a double life; an entirely independent one, belonging to its own development alone; and an incidental one, so far as it has become the constituent part of a plant. In the lowest tribes of algæ and fungi, we find these two conditions, as it were, coincident; single cells possessing in themselves the power of maintaining the life of the species, as well as their own independent vitality; or, in other words, the plant consisting of but a single cell, or being at least capable of existing as such. This is the case, for example, in the red snow (*protococcus nivalis*), in which every one of the crimson vesicles is an independent plant, having no necessary connexion with others, and capable of itself of performing all the changes required for the maintenance of its existence, and the continuance of its race. This we take to be what is expressed by the term *individuality*. The separate cells of the protococcus, however, are brought into some degree of connexion, by the mass of gelatinous matter on which they rest. The presence of this may be perceived, as a kind of slime, before any distinct traces of organization appear; and it may be regarded as standing in the same relation with the subsequently formed cells, as the organizable gummy fluid, of which we have already spoken, in the higher plants. Now this slimy substance (which has been called *phycomater*) continues to exist, in the simpler algæ, during the whole life of the cells; and, when new ones are formed by the rupture of the parent vesicle, it appears to supply them in part with their means of nutriment. On the degree in which the isolated cells are imbedded in it and connected by it, generic distinctions have been founded. Now, if we ascend to the higher forms of algæ, we shall find that the individual plants gradually become more complex in structure, and that they are composed of an association of cells having different offices in the vital economy. Still, we do not lose sight of this *organic mucus*, which, in many species, is very abundant, and which is almost always to be found between the cells. As yet, their independent life predominates over the compound life of the general structure. They live more for themselves than for each other; and there is no occasion for great intimacy in their union. In the higher plants, on the other hand, we find that the process of organization has advanced, so as to exhaust nearly all the organic mucus, and to bring the individual cells into closer contact with each other. Still, however, some traces of it may often be found; and it thus constitutes an *intercellular substance*, which may sometimes be perceived filling up interstices between the walls of the cells, and serving to unite them together. The most remarkable instance of its presence, however, is the delicate pellicle which covers the true cuticle probably of all plants, and which is even found on submerged surfaces on which no true cuticle exists. This pellicle is a very thin homogeneous membrane, in which no traces of structure can be detected. We are inclined to regard the *intercellular substance* of plants, therefore, as the remainder (so to speak) of the organic mucus out of which their structures have been organized; and to believe that this substance, lying externally to the cells, may undergo a slight degree of organization, by contact with living tissues, as we see in the case of extravasated blood, &c. We have dwelt the longer on this point, because we shall hereafter see the application of the view we have suggested to the explanation of the *intercellular substance* found in the cartilaginous and other tissues of animals. That this presents a degree of organization, can scarcely be questioned; and Schwann is perplexed to account for any structure being developed in it, without its having first passed through the form of cells.

We shall now give a short account of a few of the more important changes which vegetable cells undergo, where their ultimate function requires their form and character to be modified. One of the simplest of these modifications, which may occur in almost all the forms into which cells are changed, consists in the deposition within their walls, of certain products secreted from the vegetable juices. Sometimes these are deposited in concentric layers, exhibiting a series of rings when the cell is cut across (fig. 13). Very commonly, however, there is not this kind of regularity; but the deposits project more into the cavity. In these cases, passages are often left (as seen in fig. 14), by which the cavity of the cell is extended at some points almost to its membranous wall; and thus the different cells retain some power of communication with each other. Sometimes there is great regularity in this respect; the deposits in two contiguous cells being absent at corresponding points, so that the membranous septum is the only obstacle to the communication of their cavities (fig. 15). The object of these deposits appears to be in all instances the same—to import mechanical firmness to the tissues. They are not found to any great extent in cells which are actively contributing to the general vital processes; whilst in those which are inert, the cavity is frequently almost obliterated. Thus in the gritty tissue of the pear (fig. 16) and the stone of the plum (fig. 14), we find this *sclerogenous* deposit very abundant; and the same is the case in the woody tubes of the heart-wood of the stem; whilst those of the alburnum, through which the sap ascends, are comparatively free.

In other instances, however, the matter deposited within the cells appears to undergo organization; and it then presents the form of a spiral fibre, of which the coils are sometimes in close apposition, and sometimes widely separated. It is the opinion of Meyen that the membrane of the cell is itself composed of fibres spirally disposed; but we rather incline to agree with Schleiden that, in the cases which appear to support this hypothesis, the membrane of the cell has been originally homogeneous, and that the spiral fibres subsequently formed have become incorporated with it. The latter physiologist has seen the spiral fibre in various stages of development; and he states that its manifestation always commences at a period subsequent to that of the full development of the cell membrane. Not unfrequently the spiral fibre is afterwards broken into isolated portions by the extension of the cell; and these portions either grow irregularly together, or become the nuclei for deposits which unite them; so that an inner coat is thus formed, which is incomplete at some points, and these appear externally as dots very similar to those produced by the passages formerly described. The *dotted cells* and *dotted ducts*, therefore, may be regarded as cells or tubes within the cavities of which a secondary deposit has taken place; this deposit being sometimes entirely unorganized; whilst in other instances it seems to present some traces of the spiral structure (fig. 17).

We have not yet spoken of the alterations in *form* which the originally spheroidal or dodecahedral cells undergo at subsequent periods. In the early condition of the superior plants, as in the permanent state of the lower cryptogamia, no very great alterations of this kind ensue. At a more advanced stage of the growth of the former, however, we find several remarkable modifications. Among these are the continuous tubes or *ducts*, which seem to be the special channels for the ascent of the sap through the stem. That these are formed from large cells placed end to end, the partitions between which have been obliterated, has long been the opinion of Dr. Lindley, in opposition to the ideas of their formation entertained by the German and French anatomists. It is confirmed in a very remarkable manner by a specimen of fossil wood in our possession; the section of which, taken in a line with the ducts, exhibits most distinctly the remains of the partitions between the cells of which these tubes were originally formed. As the whole structure of these fossil woods—cavities as well as solid parts—has been infiltrated with silex, any minutiæ of this kind may be better seen in them than in the recent structures, which are liable to be torn and displaced when laid open even with the sharpest knife. This mode of formation of continuous tubes has a very interesting relation with the opinions of Dr. Hodgkin as to the origin of the absorbent vessels in animals, and the development of their valvular folds.* It is seldom that *ducts* are found composed of membrane simply; their cavities are generally kept pervious either by a spiral fibre more or less continuous, or by a wall of secondary deposit such as we have described. In fig. 17 is seen a duct, in one part of which the spiral is nearly complete, whilst in another portion it is much broken; the portions of the fibre, irregularly anastomosing together, constitute the *reticulated* duct; and when the spaces left between these have been still further closed by secondary deposits, the *dotted* duct is the result. In fig. 18 a dotted duct is seen, which seems to result from the union of cells like those represented in section at fig. 15; the inner wall, with its passages, being formed by inorganic deposit alone.

The form of those cells which remain isolated often undergoes very remarkable changes. Thus the *fibres* of woody structure, and the *spiral vessels*, are neither more nor less than very elongated cells. A class of vessels has lately been described, which bears so close a relation with the capillaries of animals that it will be worth while to enquire into their origin. These are the *vessels of the latex* or *vital vessels* of Schultz; the *cinenchyma* of Dr. Lindley. The ducts of which we have previously spoken, are all straight unanastomosing tubes. These vessels, on the contrary, present a reticulated character; and a plexus of them resembles very closely the distribution of capillaries in a frog's foot. We formerly alluded to the circulation of nutritious or elaborated sap which takes place through them;† but at that time the prevalent opinion among botanists was, that this movement takes place, not in distinct vessels, but in *intercellular spaces*. We find, however, that Dr. Lindley now avows himself a convert to the doctrine of Schultz, that there is a regular series of vessels for the performance of this function.‡ We had ourselves been previously led to the same opinion; and if the doctrines given by Schultz, for separating these vessels from the rest of the tissue, be followed, we cannot perceive any ground on which their existence can be denied. Their walls are generally very thin, and their diameter irregular. The appearance of a plexus of them in the stipules of ficus elastica is shown in fig. 19 after Schultz.

The question which we shall subsequently notice, as to the origin of the capillary vessels of animals, may not improbably be elucidated by enquiries into the mode in which these vital vessels are formed. We have ourselves no doubt that they result from the partial obliteration of the partitions between cells of irregular form, which thus open into one another in several points, and not, as when regular ducts are produced, by their extremities alone. In fig. 20 is seen a portion of cellular tissue from the stipule of ficus elastica, which seems just about to undergo this change.

We have dwelt longer on this subject than its intrinsic importance may seem to warrant, because we feel sure that the more closely the early de-

* We much regret that the valuable report on this subject, presented to the British Association at its meeting at Bristol, has not yet been published.

† Vol. IV., p. 27.

‡ Introduction to Botany. Third Edition, p. 36.

velopment of the animal and vegetable tissues is *concurrently* studied, the more will they be found to illustrate one another. Most of the details which we have now given will be found to have an important bearing on the doctrines which have been propounded by Schwann regarding the formation of the animal organism; to the exposition of which we shall now proceed. We would first remark, however, that the *increase* in the number of cells does not always seem to follow the law stated by Schleiden. In the chara, as well as in the simpler plants we have described, it is very evident that the new cells *bud out* from the sides of the parent ones, which themselves undergo no change. In the confervæ it has long been remarked that the elongation of the filaments is produced by the subdivision of the terminal cell into two, by a septum which forms across it; and the last of these in its turn undergoes a similar division. This apparent division of the cavity of cells, by the extension of septa inwards, was supposed by Schleiden to be owing to the development of two or more young cells within a parent cell and the meeting of their walls; but M. Meyen very recently,* by numerous observations, seems to have proved that such a partitioning of cells by the growth of septa really takes place even in the higher plants.

*Growth of Animal Cells.* The first animal structures, whose mode of growth Schwann investigated when he became acquainted with the discoveries of Schleiden, were the cells of the chorda dorsalis and those of cartilage. The chorda dorsalis (the gelatiniform cord which in the embryo of all mammalia occupies the axis of the spinal column in the place of the future bodies of the vertebræ, and which exists in the larvæ of amphibia and in some cartilaginous fishes throughout life,) is particularly interesting on account of the exact resemblance which its internal conformation presents to one of the most common varieties of vegetable cellular parenchyma. M. Schwann thus sums up his observations on its structure and mode of growth.

"The *chorda dorsalis* is formed of polyhedral cells. In the substance or at the inner surface of the walls of these cells lies a body which, in form, as well as position, is wholly similar to the nucleus or cytoblast of vegetable cells; since it is an oval flattened disc containing one, two, or three smaller bodies, its nucleoli. The cells lie in close contact with each other, but sometimes, at points where three or more meet, a small intercellular space or a small quantity of intercellular substance is observed. Within some of the cells young cells are formed; these are at first round and float freely in the parent cells; they do not present the characteristic nucleus, though sometimes a very minute spherical body is seen attached to the inner surface of their wall. In some instances (for example in the remains of the chorda dorsalis, which form the intervertebral bodies of osseous fishes), the cells undergo a further development. The simple membranous wall of the cell disappears, and the substance which separates the cavities of the different cells comes to be formed in chief part of longitudinal fibres." (p. 16.)

The form of the cells and of their nuclei in the chorda dorsalis of cyprinus erythrophthalmus is represented by fig. 21, plate iii. With reference to the apparent absence of the characteristic nuclei in the young cells of the chorda dorsalis (fig. 21, *b*), M. Schwann remarks (p. 17), that "it must remain uncertain whether the nucleus is really deficient

* Müller's Archiv, 1839, p. 255.

here, as at present it appears to be in many acotyledonous plants; or whether the small globule (*d*) seen on the wall of some of the young cells is a nucleus, which afterwards grows with the cell as the nucleus of some other animal cells is observed to do; or, lastly, whether the nucleus is invisible in these young cells only on account of its transparency. The last explanation is rendered probable by the circumstance that in some fully developed cells the nucleus, though certainly present, is so exceedingly translucent as to be distinguished with great difficulty." The change to a fibrous structure which the chorda dorsalis undergoes at a later period of its growth is not explained by M. Schwann.

That the corpuscules of *cartilage* discovered by Purkinje are hollow, and that they contain nuclei and sometimes globules of fat, had been noticed by several observers, namely, by Müller, Valentin, Meckauer, and Arnold. Müller and Meckauer had also shown that they might be separated as distinct bodies from the substance of the cartilage, and were not mere cavities hollowed within it, and Gurlt and Berres had even called them vesicles. Being acquainted with these facts, Schwann at once directed his attention more particularly to the phenomena of growth of the cartilages and of their corpuscules or cells.

The cartilaginous rays of the branchiæ of fishes (cyprinus erythrophthalmus), he found to be composed at their apex entirely of nucleated polyhedral cells with very thin walls (fig. 22). The nucleus was sometimes not distinct until after the action of water upon the cartilage. Towards the middle of the branchial ray the walls of the cells became evidently thicker and their cavities smaller, and it was now more manifest that each cell had distinct parietes. Moreover, at points where three or four cells met, the intervening space which was left was seen to be occupied by a new intercellular substance (fig. 23). At the base of the ray the cavities of the cells had diminished still more in size, while their walls were of yet greater thickness. The intercellular substance, too, had increased in quantity, and with it the walls of the cells had partly coalesced, so that the cartilage appeared to consist of one homogeneous mass with small excavated hollows (fig. 24). Here, then, we have an example of the walls of cells gradually increasing in thickness, a process which is not infrequent in vegetables.

This, however, is the least common form of cartilage. More usually, and in all the true cartilages of the higher vertebrata, the intercellular substance constitutes by far the greater part of the mass; while the walls of the cells retain their original tenuity, and apparently do not coalesce with the surrounding substance. The cells or corpuscules of these cartilages frequently lie in groups of three or four. M. Schwann was at first disposed to attribute this disposition of the cells to the development of several young cells within one parent cell; for in the cartilage of the branchial arches of tadpoles, it appears to him that the substance immediately around some of the groups of cells presented a concentric line comparable to the outer contour of the thickened wall of a parent cell. Further observations, however, convinced him that this view was not correct, and that the cells are arranged in groups merely from the circumstance of three or four being developed in one cavity of the intercellular substance. The figure which M. Schwann gives of a portion of cartilage taken from the margin of a branchial arch of a tadpole, illustrates extremely well the growth of cells around preexisting nuclei (fig. 25). The greater part of the mass is seen to be formed of cells with distinct parietes and with nuclei, which contain one or two small bodies (nucleoli). In the interstices of these fully-developed cells and at the free edge of the cartilage (*a*), are other cells of smaller size, in which, however, the nuclei have nearly their full dimensions (fig. 8, *c*); and between these are nuclei as yet wholly destitute of surrounding cell (*b*); so that here, as in plants, the cell seems to be gradually formed around the nucleus. Schwann also perceived here and there in the cartilage small bodies surrounded by granular matter (*e*). These were apparently nuclei in the process of development by the aggregation of granules around nucleoli, which, according to Schleiden, is the mode of formation of the nuclei in plants. Whilst the new cells are being developed by the process just described, the intercellular substance gradually increases in quantity in the interstices of those already formed, and at length constitutes the chief mass of the tissue. The solid portion of cartilage appears, therefore, to be a distinct substance, and not to be formed, even in part, by the thickening of the walls of old cells during the development of a succession of younger cartilaginous cells within them, as Schwann at first supposed. It appears certain, however, that within the proper cells of the cartilage young cells, most probably of a different physiological character, are sometimes developed from nuclei in the same manner as the cells of the cartilage were formed in cavities of the intercellular substance. This development of young cells within the proper cells of the cartilage, which is also strongly insisted on by Valentin,* has been very rarely observed by ourselves in frequent examinations of the cartilages of the human subject. But of the accuracy of M. Schwann's statement relative to the formation of the cells around their nuclei, and even of the nuclei around nucleoli, in cavities of the intercellular substance, we have had ample opportunities of satisfying ourselves.

Such are the principal facts which revealed themselves to Schwann in his investigation of the structure of the chorda dorsalis and cartilages, and which led him to infer the existence of a law regulating the development of all organic tissues, animal and vegetable. We shall now follow him in his observations on the structure and growth of animal tissues generally; and first in his account of the ovum and germinal membrane, from which all parts of the future animal are developed.

*The Ovum.* It will be recollected that the ovum of mammiferous animals, inclosed within the Graafian vesicle of the ovary, consists of three principal parts: 1, the vitelline membrane or yolk-sac; 2, the vitelline fluid; and 3, the germinal vesicle, which lies close to the inner surface of the vitelline membrane, and itself contains a smaller body, the germinal spot of Wagner. The vitelline membrane,† or external proper investment of the ovum, is, as Krause has shown, structureless, and in this character agrees with the external wall of the formative cells of plants and cartilage. The vitelline fluid also may, with very great probability of correctness, be compared to the contents of a cell. But in what light

* Repertor. für Anat. u. Physiol., i., p. 286; and Wagner's Physiologie, p. 136.

† We here leave out of consideration the question respecting the nature of the zona pellucida, since it was fully discussed in the last Number of this Journal at p. 13.

are we to regard the germinal vesicle? Is it a young cell developed within the vitelline sac, as its parent cell? Or is it the nucleus of this sac or cell? M. Schwann was at first unable to decide this question; but M. R. Wagner's observations on the development of the ova of an insect, the agrion virgo,* seemed to supply the requisite data. M. Wagner observed that the germinal vesicle was first formed, and that the vitelline sac, in its earliest condition, invested the germinal vesicle very closely, and only gradually expanded as it became filled with its contents. This fact appears to Schwann sufficient proof that the germinal vesicle is the nucleus or "cytoblast" of the ovum; with which view the position of the germinal vesicle at the inner surface of the vitelline membrane, its greater comparative size in small imperfectly developed ova, and its disappearance at a subsequent period, likewise accord. Before we were acquainted with Wagner's observation, we had ourselves noticed the same phenomena in the process of development of the ova of daphnia pulex; but a more important confirmation of Wagner's statement and of M. Schwann's deductions from it, is afforded by the discovery of Dr. Barry, that, both in birds and mammalia, the formation of the germinal vesicle precedes that of the vitelline sac. Since it appears from Wagner's drawings that the germinal spot exists previously to the vesicle which afterwards includes it, Schwann regards this spot as the nucleolus of the ovum. (*Appendix*, p. 259.)

After thus viewing the ovum as a whole, our author proceeds to examine its contents, taking for this purpose, however, the egg of the hen. We cannot follow him in his detailed description of the different kinds of globules which he found within the yolk-sac, and of the process of development of the different parts of the ovum, but must restrict ourselves to noticing the following most important facts. The yellow globules of the yolk, those of the mucous layer of the germinal membrane, and those again of the more colourless and transparent centre of the yolk (dotter-höhle), want the characteristic nucleus or cytoblast, though they contain granules. The cells of the serous layer of the germinal membrane and those investing the inner surface of the structureless vitelline membrane, on the contrary, are shown by M. Schwann to have each a nucleus of the usual form with one or two nuclei contained within it. So that in these cells formed within the ovum we have an unequivocal example of the development of nucleated cells within a parent cell. (pp. 55-66.)

The first step in the formation of the embryo consists, as is well known, in a portion of the germinal membrane becoming separated by a constriction from the rest of the vitelline sac. Both the mucous and the serous lamina contribute to the formation of the young embryo; and, in accordance with this, it is composed, according to Schwann, "of cells destitute of nucleus, and of others in which the characteristic nucleus with its nucleolus is visible. But besides these it also contains numerous nuclei free from surrounding cell." The development of the cells composing the vascular layer of the germinal membrane will come under

* Prodromus Historiæ Generationis, Lips., 1836; and Beiträge zur Geschichte der Zeugung und Entwickelung. Erster Beitrag aus der Mathematisch-physikalischen Klasse der K. Baiersch. Acad. der Wissensch. in München., 1838.

consideration at a future page, as affording an instance of the formation of new vessels.

Having thus shown that the ovum may be regarded as a nucleated cell, and that the germinal membrane and the embryo itself (the groundwork of the future animal), are composed of cells, Schwann proceeds to demonstrate that the animal tissues not only are in this general sense produced from cells, but are individually either composed of cells or developed from them by various processes of transformation. "This being true, it is evident," he remarks, "that the most scientific classification of tissues would be one based upon the different degrees of development which the cells undergo in their formation." The following is the arrangement which he proposes:

"1st Class.—Cells, of which the walls are distinct, and which remain isolated from each other. This class consists principally of the cells which float in fluids, namely, the lymph-globules, the blood-corpuscules, the globules of pus, mucus, &c.

"2d Class.—Cells, of which the walls are distinct, but which are united into coherent structures. Such are the horny tissues and the crystalline lens.

"3d Class.—Cells whose walls have coalesced. The cartilages, bone, and the ivory or proper substance of the teeth, belong to this class.

"4th Class.—Cells which have split into fibres, forming cellular tissue, tendon, and elastic tissue.

"5th Class—Cells, of which both the walls and cavities have coalesced. Muscles, nerves, and capillary vessels." (p. 74.)

1. M. Schwann has himself added no new fact to our knowledge of the structures included in his *first class*. That the *lymph-globules* are nucleated cells was proved by the observation of Vogel,* who found that, when the globule had been rendered transparent by the action of acetic acid, a darker and more opaque nucleus was visible in its interior. Of the accuracy of this observation we have convinced ourselves by the examination of the lymph-globules of the frog, in which, indeed, the nucleus can in some instances be seen before the acetic acid is added. The existence in the lymph of the frog of nuclei destitute of surrounding cell, and of small lymph-globules with nuclei, equal in size to those of the largest globules, seems to prove also that the nucleus is first formed, and that in its development, as well as in its structure, the lymph-globule accords with the true organic cells. The *blood-corpuscules* have been long regarded as cells or vesicles, and in the large elliptical corpuscles of the cold-blooded vertebrata, the existence of a nucleus has been recognized since the time of Hewson. Their mode of development, however, has not yet been satisfactorily elucidated.

The *globules of pus* and the very similar bodies poured out by the follicles of mucous membranes, have been recently shown by Vogel, Güterbock, and Valentin to contain a nucleus of different chemical properties from the rest of their substance. Vogel has observed, moreover, that in the pus-globule the nucleus is the first formed part; and that around this a semitransparent vesicle, which afterwards acquires the size and character of the perfect pus globule, is gradually developed.

We observe that Valentin† regards the blood-corpuscules, lymph-glo-

* Ueber Eiter, Eiterung und die verwandte Vorgänge. Erlangen, 1838.

† Wagner's Physiologie, p. 133.

bules and pus-globules not as nucleated cells, but as mere nuclei with nucleoli; but his grounds for this opinion appear to us by no means convincing. In the protococcus nivalis we have already seen an instance of the existence of organic cells in an isolated state in the vegetable kingdom.

2. M. Schwann's *second class of tissues* presents us with some of the most remarkable instances of the similarity of animal to vegetable structures.

Of the horny tissues, M. Schwann examines particularly the epithelium and pigment membranes, nails, hoofs, and feathers. The fact that the *epidermis and epithelium* are composed of particles of definite form, each of which contains a nucleus, is known to all anatomists; it having been recently established by the investigations of Henle and Purkinje. Henle* observed, moreover, that the particles composing the deepest layer of the epidermis, or the rete mucosum, are globular and so small in size as to be nearly filled by their nucleus; and that, whilst the nuclei retain nearly their original size, the cells constituting the more superficial layer are larger as well as flattened. These facts are confirmed by Schwann, who points out their complete accordance with his theory. He remarks, also, that when the cells of epithelium have the globular or polyhedral form, as on the branchial rays of fishes, the nucleus can be seen to be attached to the wall of the cell. In the tadpole, Schwann has seen young nucleated cells within cells of the epithelium. (pp. 82-7.)

The *pigment membranes*, for example, that of the choroid coat of the eye, are known to be composed of polygonal cells, the centre of each of which is occupied by a colourless nucleus, and the surrounding space by granules of pigment. Schwann has not any observations upon the mode of growth of these cells; but we find Valentin stating† that the nucleus is first formed, that a cell is developed around it, enlarges and becomes polyhedral, and that subsequently the deposition of pigment molecules within the cell commences around the nucleus.

The transformation from the globular to the radiate form, which pigment cells sometimes undergo, displays to us, as we shall presently see, the type upon which capillary vessels appear to be developed.

The investigation of the structure of the *nails* in the fœtus and new-born infant afforded to Schwann a fresh confirmation of his theory. He found that the thin horizontal laminæ, into which the nail may be split or torn, were not structureless, but were composed of scales like those of epithelium, and in some of the scales he could distinguish a nucleus. The root of the nail, particularly at its under surface, presented no laminated structure, but consisted wholly of small polyhedral cells, very many of which had a distinct nucleus. Hence, the growth of the nail would seem to consist in the formation of cells, which subsequently become flattened and extended. But since, if this process went on only at its root, the nail, while it would be pushed forwards, would become thinner from behind forwards, in proportion to the flattening of the cells, it appears necessary, as M. Schwann remarks, that the formation of new cells

* Symbolæ ad Anatomiam villorum intestinalium, &c., p. 5. Berol., 1837.
† Wagner's Physiologie, p. 135.

should take place likewise at the under surface of the nail, at least to a certain extent from its root. (pp. 90-2.)

The horny tissues of the cloven *hoofs* of ruminantia was found by M. Schwann to be entirely composed of polyhedral cells; and in some of those most recently formed he was able to recognize a nucleus attached to the inner surface of their wall.

The medullary substance of *feathers* (described by Hooke,* and both described and figured by Leeuwenhoek† as consisting of globules or vesicles), presents the most exact resemblance to the cellular parenchyma of vegetables; and its mode of growth also, according to Schwann, agrees very closely with the process described by Schleiden as obtaining in plants. The development of animal cells is, indeed, nowhere more easily observed than in the young feather, at the period when the medullary substance of the shaft is in the process of formation. The surface of the matrix is then covered with a finely granular matter containing numerous small nuclei, some of which have a nucleolus (fig. 26). At a little distance from the surface of the matrix each nucleus is surrounded by a cell which is at first not much larger than the nucleus, and has a granular aspect (fig. 27, *c*, *b*). The further advanced the medullary substance is in its formation, the larger are the cells; and the nucleus also enlarges to a certain extent, but remains attached to one wall of the cell (fig. 27, *a*). While the cells increase in size, their walls become thinner and acquire a darker contour. At length they adhere together, and assume a polyhedral form, at the same time that the nuclei disappear (fig. 28). (*Schwann*, pp. 94-7.)

We shall reserve M. Schwann's account of the development of the cortical part of feathers, which is a fibrous structure, until we come to speak of his fourth class of tissues.

In the foregoing review of the growth of the horny structures, we have met with only two instances of the cells undergoing a remarkable transformation, and these we have reserved for future consideration, while generally the cells have preserved a resemblance to the simplest forms of vegetable tissue. But in the *crystalline lens*, which M. Schwann places in the same class with those structures, we can at first perceive nothing which tends to confirm the correctness of that classification, or which even indicates in any way the origin of the tissue from cells. Yet M. Schwann's observations lead him to conclude that the crystalline lens, so far from affording a contradiction, presents appearances remarkably favorable to the general theory.

The lens, as is well known, is composed of concentric laminæ, each of which is constituted of fibres united to each other by tooth-like processes of their margins. It was some time since, however, observed by Werneck,‡ and confirmed by Ahrens,§ that a layer of transparent cells intervenes between the lens and its capsule; and from such cells the fibres of the lens, according to Schwann, are formed. In a chick, after eight days' incubation of the egg, he found the lens to consist entirely of round, extremely pale, transparent, and smooth cells, some of which were furnished with a nucleus, mixed with nuclei destitute of surrounding cells

* Micrographia, p. 165. † Arcana Naturæ. Epistola 74.

‡ Ammon's Zeitschrift. Bd. v. p. 414. § Müller's Archiv, 1838, p. 259.

(figs. 29 and 30). In the eye of a fetal pig three inches and a half long, the lens, as yet only partly formed, was imbedded in numerous cells similar to those above described, while its circumference was formed by a thick and broad zone of imperfectly developed fibres evidently in the stage of transformation from cells. These imperfect fibres, like the perfect ones, formed arches from the anterior to the posterior surface of the lens, but neither in front nor behind did they reach its axis, while their extremities were either abruptly truncated, or slightly dilated, or expanded into a vesicle or cell, which resembled exactly the free cells on their exterior (fig. 31). These appearances seemed to show that the fibres of the lens are formed by the elongation of pre-existing cells—a view confirmed by the fact that nuclei were often seen by M. Schwann within the fibres of the lens of the fetal pig. The cells which have thus assumed the form of fibres seemed to acquire gradually the dentated margin; for they had that character in a more marked degree in proportion as they were examined nearer to the centre of the lens. The dentations of the fibres of the lens are compared by M. Schwann to the sinuosities of a not uncommon form of vegetable cells. (pp. 99-102.)

Valentin had as early as 1833 suggested that the cells discovered by Werneck were converted into the fibres of the lens. The process, however, by which he now describes that conversion to take place, is different from the one observed by Schwann. Several cells, he says, coalesce in a longitudinal series, and then split into fibres.* Schwann several times observed a linear arrangement of nuclei, but saw nothing which seemed to indicate that one of the fibres of the lens was formed by the coalescence of several cells.

3. Schwann's *third class* of tissues, to which he refers the cartilages, bones, and teeth, appears to us to be the least natural of all, or rather, perhaps, we should say that Schwann's investigation of those structures was too imperfect to justify his including them under one definition. He designates them as tissues in which the cells have coalesced either with each other or with an intercellular substance. But we have already seen that in the cartilages of the higher vertebrata the walls of the cells remain distinct. Schwann perceived this, and remarks that here the presence of a solid intercellular substance must be taken as the characteristic of the tissue. In the teeth, however, we shall find that the intercellular substance appears to be wanting.

We have previously described the development of the cells of *cartilage*, which accords so entirely with Schwann's theory: it, therefore, merely remains for us here to mention a circumstance in the history of this tissue, which to Schwann appeared anomalous, but of which we shall presently offer an explanation. It is the gradual conversion, as age advances, of the intercellular substance, which in the fœtus and young child is perfectly homogeneous, into a structure composed of distinct parallel fibres. This change is not universal, nor does it take place to the same extent in all parts of the same mass of cartilage; but yet it is frequently very marked. Moreover in one kind of cartilage, that composing the external ear and epiglottis, the intercellular substance is at all periods composed of minute fibres.

* Wagner's Physiologie, p. 138.

The osseous corpuscules and the intervening substance of *bone* seem to be identical with the corresponding parts of cartilage, which in the process of ossification become impregnated with calcareous salts, while the animal matter undergoes, as Müller has shown, a chemical change. We have, therefore, only to notice Schwann's mode of explaining the formation of the calcigerous canaliculi which radiate from the osseous corpuscules. There are two processes, he says (p. 35), by which they might be developed. They might arise from the walls of the cells undergoing thickening by deposition on their internal surface, except at certain points, where pore-like canals would be left, a process which is observed to take place in vegetables (*see* figs. 14, 15, and 16, plate i.); or they might be produced by the elongation and branching of the cells in the manner in which we shall see that certain ramified pigment cells, and perhaps also the capillary vessels, are formed. M. Schwann prefers the latter explanation, and probably with reason; but when he states that there is no instance in the animal organism of pores being left during the thickening of the walls of cells, as in the formation of the dotted cells and ducts, and other similar structures of plants, he is at variance with Valentin,* who describes such a process as taking place in the organization of the external skeleton of the astacus fluviatilis.

In the growth of the *teeth*, and even in that of their enamel, M. Schwann finds some confirmation of his theory. He has ascertained that the hexagonal fibres of the enamel membrane of Raschkow, described at p. 167 of the Eighth Volume of this Journal, are elongated cells (fig. 33). Each of them contains a distinct nucleus, while the membrane upon which they rest is also formed in great part by round nucleated cells, apparently the primary form of the above-mentioned fibre-like prismatic cells. Moreover, the mass of organic matter left, when the enamel of the young tooth of a child or pig was submitted to the action of dilute muriatic acid, was found by Schwann to consist of closely-aggregated prisms (fig. 32), exactly similar to the fibres of the enamel membrane. Accordingly he supposes that, by a process of transformation constantly going on at the surface of the enamel membrane, the globular cells of that membrane are changed into prismatic fibres or cells, which immediately separate from it, and, being at the same time filled or impregnated with calcareous salts, become part of the growing enamel. (pp. 118-21.)

The ivory of the tooth, or at least its intertubular substance would seem to be formed by a somewhat similar process. The globules spoken of by Purkinje and Raschkow as composing the formative pulp of the tooth, are, according to Schwann, round nucleated cells; while the elongated globules, described by those anatomists as being more regularly arranged at the surface of the pulp with their long axis directed outwards, are cylindrical cells, which, like the prismatic cells of the enamel membrane, contain nuclei with their nucleoli. When the pulp is drawn from the cavity of a young tooth, a layer of these cylindrical cells remains attached, at all events in some parts, to its inner surface. These facts at once led Schwann to imagine that there was a direct conversion of the cells of the pulp into the dental substance—in other words, that "the tooth was the ossified pulp." (p. 124.) But it appeared at first doubtful,

* Repertorium für Anat., Bd. i., p. 124; and Wagner's Physiologie, p. 135.

in what relation the ossifying cells stood to the different parts of the perfect ivory. Purkinje and Raschkow, however, had stated that the ivory of the growing tooth consisted of fibres directed from the pulp to the outer surface; and Schwann, examining these fibres (fig. 34), found that, while they corresponded in size to the elongated cells above mentioned, they were much larger than the now well-known dental tubuli. Hence he concludes that the cells of the pulp, becoming filled with animal matter and then ossified, form the fibres of Purkinje and Raschkow, and that these coalesce so as to constitute the intertubular substance of the tooth leaving, however, spaces in which the dental tubuli are at a subsequent period developed (pp. 122-8). This view of the mode of growth of the ivory accords very well with the observation of Mr. Owen* and Mr. Nasmyth,† that, even in the perfectly ossified tooth, cells can be distinguished in the intertubular substance. The ivory, therefore, would appear to contain no element corresponding to the intercellular substance of cartilage.

4. We now pass to the consideration of the tissues of the *fourth class* —those produced by the elongation of cells.

In these fibrous structures, the cellular tissue, tendon and elastic tissue, all trace of the original cellular form is lost; and yet it is here that the testimony of other observers has afforded perhaps the most abundant confirmation of M. Schwann's researches.

The essential elements of *cellular tissue* are now generally admitted to be wavy bundles of cylindrical fibres, between the interlacements of which are deposited in some parts the cells or vesicles of fat. When, however, cellular tissue is examined in the young embryo, it is found to be a gelatiniform substance, composed of a transparent base containing more or less numerous corpuscules of various kinds. The most constant of these corpuscules are the true primary cells of the fibres of cellular tissue. In their earliest state, these cells are round, and possess the characteristic nucleus, with one or two nucleoli (fig. 35). It seems probable, indeed, that the nucleus exists prior to the cell, and that the latter is gradually formed around it; for M. Schwann observed many nuclei without investing cell, but no cell without a nucleus. When further developed, the nucleated cells become acuminated at two opposite points (fig. 35, *b*); then they appear drawn out at these points into fibres, which are sometimes branched (as at *a*); and at a more advanced stage these fibres resolve themselves into bundles of fibrils (fig. 36). The division into fibrils commences, according to Schwann, at the extremities of the fibres, and gradually advances towards the body of the cell, which at length becomes involved in the change. The nucleus remains visible for a time, lying upon the fasciculus of fibrils, but is afterwards absorbed (pp. 135-9). The accuracy of these observations of M. Schwann seems to be placed beyond a doubt, by the circumstance to which we have before alluded, that similar facts have been described, though in different language, both by earlier and by contemporary writers. R. Froriep had in 1837‡ remarked that the exudations of pericarditis contained

* Report of the British Association for the Advancement of Science, 1838, p. 140.

† Meeting of British Association, 1839. Athenæum, No. dcxx.

‡ Klin. Kupfertafeln, 11te Lief., Th. lxi.—Weimar, 1837.

fibres similar to those of cellular tissue, and also irregular granules, which appeared to be elongated into thin threads in one or two directions. "These elongated fibrinous granules," he adds, "seem to be the first form of the nascent cylindrical fibres of the true pseudo-membrane or cicatrix." Güterbock,* and Valentin† likewise, had observed globules and spindle-shaped bodies, mixed with fibres, in the exudations of inflamed serous membranes and in granulations. Appearances confirmatory of Schwann's doctrine of the mode of development of cellular and fibrous tissue generally, have since been witnessed by Henle, Müller, Breschet, and Simon. In condylomata, in which Simon‡ observed the growth of the cylindrical fibres, we have also had the opportunity of seeing the various forms intermediate between the round nucleated cell and the cylindrical fibre. There is some discrepancy, though none affecting the validity of the general theory, between Valentin's more recent account§ of the development of the fibres of cellular tissue and that given by Schwann. According to Valentin, the primary cells arrange themselves in a linear order, become elongated, and then coalesce, so as to produce at first knotted fibres, the enlargements of which are due to the nuclei of the original cells. By the gradual elongation of these fibres, and the absorption of the nuclei, uniform cylindrical fibres are produced, which subsequently resolve themselves into smaller fibrils. Neither Schwann nor any other minute anatomist appears to have observed this coalescence of cells in a linear series in the process of development of the cellular and allied tissues. There is, however, a tissue investing the minute parts of glands and other organs, which, in the knotted or nucleated form of its fibres, seems to represent in a permanent condition the transitory stage pointed out by Valentin in the growth of the cylindrical fibres of cellular tissue.

Mingled with the primary cells of the cellular tissue, in the embryo, Schwann found also *cells of adipose matter.* They were round vesicles, of different sizes, containing generally one globule of fat which completely filled them. In their wall, which was finely granular or structureless, Schwann recognized a distinct nucleus (p. 140). This nucleus disappears at a later period; but, according to Valentin,|| is not absorbed, for he was able to distinguish it in emaciated persons, in whom the adipose cells contained only a few granules of fat.

Schwann observed a third kind of cell in the cellular tissue of the fœtus, the nature of which, however, he could not determine.

The fibres of *tendon* are developed by the same process as those of cellular tissue, which they so closely resemble (p. 147).

M. Schwann's investigation into the mode of growth of *elastic tissue*, though it satisfied him that there was here no exception to the general law of development from cells, yet did not reveal the exact steps by which the cells are transformed into the elastic fibres. The appearances, however, which he observed (fig. 37), tended to show that in this tissue the fibres are produced not so much by the elongation as by the

* De pure et granulatione.—Berol., 1837. † Repertorium, 1837; p. 259.

‡ Müller's Archiv, 1839; p. 26.

§ Repertorium für Anatom. u. Physiol., 1838, p. 309; and Wagner's Physiologie, p. 137.

|| Wagner's Physiologie, p. 135.

expansion and subsequent splitting of the body of the cell (pp. 148-51). Should this prove to be the case, the fibres of elastic tissue would, Schwann observes, hold a middle position, as to their mode of development between the fibres of cellular tissue and tendon, and those composing the cortical structure of feathers.

The cortical substance of the shaft of feathers belongs to the horny structures; but it appeared to us that M. Schwann's observations on its mode of development would be read with greater interest, if placed in connexion with his remarks on the other fibrous structures.

Examined in the imperfectly formed feather, the cortical substance of the shaft is found to be composed of large, flat, somewhat granular laminæ or cells, each of which contains a distinct nucleus, with one or two nucleoli (fig. 38, *a*). An indistinct appearance of longitudinal fibres is observed at the borders of the laminæ in parts more advanced in development. These fibres gradually become more defined, and may be traced through the whole extent of the cell (fig. 38, *b*). At the same time the nucleus begins to be absorbed, and last of all the nucleolus disappears (fig. 38, *c*, *d*). When perfectly developed, the fibres produced by the resolution of the different laminæ or cells become continuous with each other (pp. 97-8). Here, then, no elongation of the cells takes place, and we have an example of the transformation of cells into fibres by mere division.

It seems very probable that the fibres composing all the tissues of this class are really hollow cylinders; for in a form of pigment tissue, which we shall presently describe, the threadlike prolongations of the cells are certainly tubular, the dark colour of the matter contained in them rendering it visible; and Purkinje and Räuschel have seen indications of a cavity in the fibres of elastic tissue. If such is the case,—if the fibres of the cellular, elastic, tendinous, and horny tissues are hollow,—then the first step in the division of their primary cell must consist in the growth inwards of linear septa, and, though the form of tissue resulting is very different, an essential analogy must be recognized as subsisting between the process of the production of these animal fibres and that of the multiplication of cells in vegetables by the growth inwards of septa from the walls of the primary nucleated cells, to which we have already referred.

5. The fifth class of tissues—those which result from the coalescence of the cavities, as well as of the walls of cells—includes the most important and highly-endowed elements of the animal organism.

There are two principal types according to which these tissues are formed. In the one case, the primary cells arrange themselves in a linear series, and then coalesce, so as to form a cylindrical tube, denominated by M. Schwann the secondary cell; in the other, the cells not arranged in a regular order first assume a stellate form, and being at considerable distances from each other, coalesce only through the medium of their radiate processes. The latter is the mode of formation of the reticulated capillaries; the former is the type after which muscles and nerves are developed. We have already described the formation of ducts and other vessels in plants by the same process,—the coalescence of cells and the subsequent absorption of the septa which at first separate their cavities.

The first steps in the development of *muscular fibre* have not been observed by Schwann. Long since, however, Valentin had remarked that, before fibres can be distinguished in the place of the future muscle, globules (probably primary cells) are seen, arranged in longitudinal lines; and that these globules gradually coalesce, so as to form first beaded and then cylindrical threads. The principal facts ascertained by Schwann, relative to the subsequent steps in the process, are as follows: In the fibres which have acquired the cylindrical form, nuclei are still visible. While the fibres are irregular on their surface, the nuclei within them lie very close to each other, and, on account of the granular matter surrounding them, are indistinct (fig. 39, *a*, *b*, *c*). They are rendered very evident, however, by the action of acetic acid (fig. 40). Afterwards, as the fibres become elongated, and somewhat narrower, and lose their granular aspect, the nuclei begin to be more widely separated, and themselves assume a lengthened form. The fibres are now distinctly hollow, and the nuclei lie in the substance of their walls (fig. 41, *a*, *b*, *c*). The cavities of the primary cells have at this period coalesced, so as to form the secondary cell or tubular fibre. A deposition of new matter takes place on the inner surface of the secondary cell, and continues until the cavity of the cylinder is quite filled; and, subsequently, this new matter is converted into the longitudinal muscular fibrillæ which compose the striated muscular fibre. The transverse striæ become visible in the human embryo, according to Valentin, about the sixth month. The nuclei of the primary cells gradually disappear; but the membranous wall of the cylinder is believed by M. Schwann to exist throughout life (p. 156-66).

*The Nerves.* Schwann has not satisfactorily seen the primary cells from which the nervous fibres are developed. The nerves, in the earliest condition in which he examined them (in the fetal pig), "appeared as pale, finely-granulated cords, in which indistinctly-shaded lines gave some indications of a coarse fibrous structure. Through the whole thickness of these cords, and principally in the course of the shaded lines just mentioned, nuclei, containing one or two nucleoli, were thickly scattered (fig. 42). Occasionally, a single fibre separated itself from the nerve in a part of its extent (fig. 42, *a*), and it then became evident that the nuclei belonged to the fibres, and that several were contained in one fibre" (p. 171). These fibres, which Schwann supposes to be hollow, and to be produced by the coalescence of several primary cells, have a pale, granular aspect, and want the dark outline of the perfectly-formed elements of the nerves (figs. 42, *a*, and 43, *a*). But their identity with the nervous fibres seems certain; for, in nerves further developed than those above described, Schwann found, mixed with the pellucid granular substance, distinct nervous fibres similar to those of the adult animal, and, beside these, other fibres in which the transition of the imperfect nervous fibres into the perfect ones was evident (fig. 43, *b*). The mode in which the transformation is here effected is not so clear. The dark outline of the nervous fibre is due to the same substance which gives them their white colour; and Schwann inclines to the opinion that this substance is deposited, in the same manner as the matter which forms the ultimate fibrils of muscular fibre, on the inner surface of the tube resulting from the coalescence of the primary cells. The only observation, however, which he adduces in support of his opinion is, that in nervous fibres of the adult animal a delicate, structureless, transparent membrane exists on the exterior of the white substance. Once or twice, indeed, he observed appearances (fig. 44) which led him to suppose that the nuclei of the primary cells sometimes remain for a considerable time unabsorbed in this transparent sheath of the nervous fibre (pp. 171-5). But, on this as well as on many other points relating to the development of the nerves, M. Schwann's observations are less satisfactory than his account of the process by which the muscular tissue is formed.

The globules of the nervous ganglia and gray substance of the brain and spinal cord are evidently nucleated cells within which a new substance has been deposited. Schwann has no observations on their mode of growth; but Valentin* has observed that the nucleus is formed first, and that the cell is afterwards gradually developed around it.

The growth of *the capillary vessels,* though a process of such paramount importance in the animal economy, and one which is almost constantly offering itself to our observation amongst the sequels of injuries or disease, has nevertheless baffled, hitherto, the endeavours of the numerous physiologists and pathologists who have laboured to elucidate its nature. Most of the hypotheses which have been framed to explain it have been far too fanciful and mechanical in their character. Schwann's observations on this subject are, confessedly, imperfect; but yet they appear to us so interesting, that we shall give a tolerably copious abstract of them.

Schwann prefaces his remarks on the capillaries themselves by a reference to a particular form of pigment cell which is observed in the skin of the batrachian amphibia. The dark spots in the skin of these animals are dependent on the presence of bodies, chiefly of a stellate and ramified figure, containing pigment granules (fig. 46). Schwann shows that these are really cells; that every stage of the transition can be observed by which ordinary round or polygonal pigment cells with nuclei acquire the radiate form (fig. 45, *a*, *b*, *c*, *d*, *e*, *f*); and that even in their most developed condition each of the ramified cells contains a nucleus. Such are the cells of which Schwann speaks in the following passage:

"At fig. 46 is a representation of two stellate pigment cells which have coalesced at *a*........... At the point where the arms of the two cells unite, their cavities seem to communicate; at all events, there is no apparent interruption to the line of pigment which they contain. Now, if we imagine several such stellate cells to be developed in a surface of considerable extent at similar distances from each other, and if we further suppose the several prolongations sent out by each cell to coalesce with those sent out by the other cells, we shall have an extended network of continuous canals. The size of the meshes in such a network would be determined by the distance of the original cells from each other, and by the number of the prolongations proceeding from each cell. Such appears to be the process by which capillary vessels are formed." (pp. 182-3.)

The observations which led Schwann to believe that the capillaries

* Wagner's Physiologie, p. 135.

are developed by the process described in the above extract, are the following:

"1. The walls of the capillaries in the tail of the tadpole are formed by a distinct though delicate membrane. . . . . .

"2. Nuclei similar to those of primary cells can be distinguished in the walls of the capillaries of the tadpole. . . . These nuclei are not sufficiently numerous to belong to scales of epithelium investing the inner surface of the vessels; and it is, therefore, more probable that they are the nuclei of primary cells, by the coalescence of which the capillaries have been formed.

"3. In the tail of very young tadpoles the capillary network presents, besides the ordinary cylindrical canals in which the blood flows in a regular current, other vessels of an irregular form. . . . . These vessels agree in essential characters with those which I shall presently describe, as found in the germinal membrane of the egg, where, however, the meshes are not nearly so large. The irregularly-formed capillaries to which I refer are usually widest where branches are given off (as at the points *a* and *b* in the diagram, fig. 47), but taper very rapidly as they leave those parts, and then again widen until they form a second dilated part. They present every possible degree of narrowing, from that which would scarcely be remarked, to that where the vessel is reduced to a thread not much thicker than a fibre of cellular tissue (as at *c*). Moreover, from the wide parts of these varicose vessels branches sometimes proceed, which gradually diminish in size, and are lost without reaching another broader part of the network (as at *d* and *e*). According to the view of the development of capillaries which I adopt, these appearances may be explained in the following manner: The wide parts of the vessels represent the bodies of the primary cells. These have sent out in a stellate form hollow processes, which have in most parts met and coalesced with similar processes from other cells. These processes, however, being hollow, are able to dilate during their growth, and consequently can assume the form of the ordinary cylindrical capillaries (as at *f* and *g*). . . . That the vessels here described are blood-vessels, is proved by their direct continuity with the ordinary capillaries, and also by the wider of them being actually entered by blood corpuscules. . . . . To prove, however, that they are capillaries in the stage of development by the process we have imagined, it is necessary to show the existence of the primary cells before their union with the actual blood-vessels. . . . . Stellate cells certainly exist in the same stratum with the capillary network in the tail of the tadpole, and processes from these cells sometimes appear to unite with prolongations of the capillaries. The reality of these anastomoses, however, is not quite certain; and the great number of the stellate cells, and their existence at all ages of the tadpole, are circumstances unfavorable to the supposition that they are primary cells of capillaries. . . . . The uncertainty which surrounds this point appears, however, to be removed, when our observation is transferred to the incubated egg.

"4. The germinal membrane of an egg which has been subjected to thirty-six hours' incubation being examined with a microscopic power of 450 diameters, the capillary vessels are readily distinguished in it by their yellowish-red colour. . . . In some parts the vessels are perfect; in other parts they have the imperfect form described above as it was seen in the tadpole. In addition to the vessels, however, there are seen here and there in the germinal membrane bodies of irregular form which are not connected with the vascular network. These bodies give off blind processes in different directions, and hence have the appearance of star-shaped cells (fig. 29, *h*, *i*). Their colour is yellowish-red, like that of the true capillaries; which fact is alone sufficient to suggest the suspicion that they are an early condition of those vessels. And this suspicion is strengthened by the fact that some of these bodies (such as *k*) seem to be actually connected to branches of the capillaries. We may, therefore, at least, with a high degree of probability, regard them as primary cells in the act of becoming transformed into capillaries by the process above indicated. . . . . The fluid portion of the blood constitutes the contents of the primary cells as well as of

**the vessels produced by their coalescence; and the blood corpuscules are probably young cells developed within the cells which have united to form the network." (pp. 183-8.)**

Having thus followed our author in the detail of his anatomical researches, let us examine how far they justify the expectation with which he set out, namely, that one general law would be found to regulate the development of all organic elements, animal as well as vegetable, how different soever their form and physiological endowments. The observations of Schleiden had demonstrated the process of growth of the elementary parts of plants: they had shown that in all cases a body of peculiar conformation is first produced by the aggregation of molecules of a preexisting structureless substance, that a cell is gradually developed around this body, and that the cell subsequently undergoes various modifications, according to the kind of tissue which it is destined to form. The task which Schwann undertook was to ascertain whether this process obtained also in the growth of animal tissues. To prove to demonstration that the same law of development prevailed *universally* amongst organic structures was scarcely possible; but Schwann perceived that the principle would be sufficiently established, if *the greater number* of animal tissues essentially distinct in their physiological properties could be shown to agree with each other and with the vegetable tissues in the process of their formation, and if, on the other hand, no tissue should appear to be developed by a distinctly different process. Viewed in this light, the facts adduced by M. Schwann afford an adequate basis for the theory which he has raised upon them. He shows, 1, that, in all the tissues which he has examined, the presence of a body wholly similar to the cytoblast of vegetable cells may at an early period of their existence be demonstrated; 2, that structures the most various—the ovum and a part of its contents, the globules of the animal fluids, the epithelium and other horny tissues, the crytalline lens, cartilage, fibrous tissues and muscle,—either are produced by the transformation of cells containing each a nucleus (which, like the cytoblast in plants, lies in the substance of the wall of the cell), or permanently retain the form of nucleated cells; and 3, that, in the growth of many structures differing entirely in their form and functional properties,—namely, the ovum, pus-globule, horny tissues, lens, cartilage, and cellular tissue,—the nucleus or cytoblast exists before the cell, just as in the process demonstrated by Schleiden in plants.

Schwann acknowledges that there are one or two apparent exceptions to the law that all tissues are developed from cells. One of these, and the most striking one, we have already noticed, namely, the conversion of the intercellular substance of cartilages into a fibrous structure. But though this instance proves that fibres may be developed in other ways than by the elongation and splitting of cells, yet far from regarding it as a formidable objection to the general theory, we are led by the following reasons to refer the formation of the fibres even here to the agency of cells: In the first place, from the history of the development of cartilage it may be inferred, that its corpuscules or cells are the essential agents in the formation and maintenance of the tissue, and that the solid intercellular substance is a secondary and dependent part. Secondly, in the fibrillæ of muscular fibres, and in the spiral fibres of the spiral vessels of plants, we have examples of a deposit within cells assuming the form of fibres; and thirdly, since, as we shall presently show, the walls of the cells can certainly produce chemical changes in the matter around them, it appears to us possible that, in the case of the cartilage, they may also diffuse such a plastic influence on their exterior, as in muscle and the vegetable tissues they seem to exert on the depositions within their cavities.

We have already mentioned, incidentally, several observations by other microscopic enquirers which aid in confirming M. Schwann's theory. The labours of Müller, however, being much more comprehensive and important, demand a more especial reference. In his late work on the microscopic structure of morbid growths,* he not only shows the presence in the various diseased structures of nucleated cells wholly analogous to those of the healthy animal and vegetable organisms, but he demonstrates the growth of these cells from nuclei and the development of young cells within parent ones, and points out that the caudate or spindle-shaped bodies, previously noticed by him and others in morbid tumours, are nucleated cells in the stage of transformation into fibres. Müller, indeed, states that there are no microscopic elements of diseased growths which may not be referred to one or other of the various forms which cells assume in the progress of their development into the different natural tissues. Many facts have been observed by Valentin,† also, in the field of morbid anatomy, which accord with Schwann's proposed law. Again, the bodies described by Dr. Hake‡ as constituting the basis of a peculiar disease, met with hitherto only in the liver of the rabbit, are a striking example of morbid nucleated cells, which, as we have ascertained, present in their mode of development no exception to Schwann's law; their nucleus being first formed, and the cell gradually produced around it. The operations of disease, therefore, in the building up of abnormal structures, obey the same general laws which regulate the nutritive processes of the frame in its healthy state.

In the preceding pages our principal aim has been to lay before our readers a faithful analysis of the first and second sections of M. Schwann's admirable work. We have shown the steps by which he was led to infer the existence of an important law, and have given a succinct, but we hope intelligible, statement of the facts which convinced him of the accuracy of his previous reasonings. Another object, however, has been kept in mind, namely, by bringing together the observations of other recent observers, to present a general view of the existing state of knowledge respecting the development of the elementary tissues.

The third and last section of M. Schwann's treatise is occupied partly by a review of the process by which the observations of Schleiden and himself had shown the organic tissues to be developed, and partly by an enquiry concerning the proximate cause or force upon which that process, and vital phenomena generally, depend. It will not be necessary for us to analyze closely the former part of this section. There are, however,

* Ueber den feineren Bau und die Formen der krankhaften Geschwülste. 1ste Lief.—Berlin, 1838.

† Repertorium, 1837 and 1838. ‡ On carcinoma of the hepatic ducts, &c., 1839.

one or two particulars connected with the morphological history of the organic cells, which we have as yet scarcely touched upon, but which, since they are of interest and importance, we must briefly notice. We shall then enter upon the consideration of the active properties of the cells, and of their relation to the vital phenomena of organized beings.

One of the points in the morphology of the cells to which we have alluded respects the situation in which new cells are developed in relation to those already existing. In plants, according to Schleiden, the young cells are always formed within fully-developed parent cells, and never on the exterior of the old cells. In the animal organism the contrary appears to be the rule; the formation of the new cells generally takes place on the exterior and in the interstices of the old ones, though here also there are instances of the growth of cells upon nuclei within parent cells. Thus, Schwann has observed it to occur occasionally in cartilage and in the chorda dorsalis; and the ovum, he remarks, affords a distinct instance of the formation of numerous cells within a parent cell. The observations of Dumortier, also, upon the development of the ovum of gasteropoda (published previous to M. Schwann's researches),* seem to show that in those animals the liver is formed by the successive development of cells within cells. In diseased growths, too, Valentin and Müller, as we have before mentioned, have observed a similar process.

Another circumstance worthy of remark is that of the situation in which the new cells are developed in relation to the mass of the tissue. The formation of the new cells takes place in a fluid, gelatinous, or even solid substance, which lies either in the interior or, more usually, in the interstices of the existing cells. This substance, called by Schwann the "cytoblastoma," affords the matter for the formation of the cells; but, to enable it to perform this important office, it requires to receive constantly fresh supplies of nutritive matter from the blood-vessels. Hence arises the difference between the mode of growth of vascular and that of non-vascular tissues. In vascular tissues the nutritive fluid—the liquor sanguinis—permeates every part, and new cells, which are afterwards transformed into the elements of the tissue, can consequently be developed throughout their substance. But non-vascular tissues, such as the epidermis, being accessible to the nutritive fluid at that surface only where they are in contact with a vascular tissue, the addition to their substance by the formation of new cells can take place only in that situation. This constitutes the sole essential difference between vascular and non-vascular tissues. Both are organized; in both the elements or cells grow by spontaneous assimilation. We now proceed to speak of the active properties of the cells, of which assimilation is only one of the results.

The phenomena presented by the cells during their life within the organism may be referred to two natural groups, denominated by Schwann the *plastic* and the *metabolic* (from *τὸ μεταβολικον*, that which is prone to cause or suffer change.)

The *plastic* phenomena are those which we have already studied in so extended a manner, as displayed in the development of the different tis-

* Annal. d. Sc. Nat., t. viii., p. 129.

sues, namely, the aggregation of molecules of organic matter in accordance with a definite plan, so as to form successively the nucleolus, nucleus, and cell; the transformation of the cells into the elements of the various tissues; and the deposition of a new substance within the simple or compound cells. These phenomena may be dependent on a force of attraction resident in the nucleoli and nuclei of the cells, as well as in the cells themselves; but it is evident, as M. Schwann remarks, that with this some other power must be combined. For althoughthe cytoblastema contains all the elements from which the component matter of the cells is formed, they exist in it in different combinations. The cells, therefore, must have the power not only of attracting, but also of chemically changing the substances brought into contact with them (p. 234). The phenomena to which this power gives rise are the *metabolic* phenomena; and the power itself is called by Schwann the *metabolic force.*

An interesting example of the action of this force, and one which proves its seat to be in the cells and not in the surrounding matter, is pointed out by Schwann in the process of vinous fermentation. The recent discoveries respecting the nature of fermentation are described in another part of this volume (see p. 579); but the organic cells, on the agency of which the process depends, are represented by fig. 12, *a, b, c.*

A remarkable instance of the metabolic action of cells in the animal organism is presented to us in the change of composition which the substance of cellular tissue undergoes during its development. In its imperfect condition, when the cells are in process of formation, this tissue has been found by Güterbock,* Schwann, and Simon,† to contain pyine, or a substance nearly allied to that principle, and no gelatine, which is well known to be its chief component in its perfect state. During the process of ossification, a parallel change takes place in the animal matter of cartilage. The principal animal constituent of unossified cartilage is chondrine; that of bone is true gelatine. An anatomical fact also of great interest in relation to the chemical action of the cells has been pointed out by Schwann,‡ Purkinje,§ and Henle.|| They have shown that the essential component elements of all glands are nucleated cells, sometimes disposed as an epithelium-like layer on the interior of tubular canals, at other times united in masses, so as to form nearly solid acini. It cannot be doubted that these cells are in some way connected with the action of the gland, and it is most probable that they are the efficient agents in the secreting process.

We have hitherto spoken of the metabolic phenomena merely as the results of an action of the cells by which they decompose the organic matters in contact with them. But such an action will not explain all the metabolic phenomena of the cells. "The great difference which frequently exists between their contents and the surrounding cytoblastema seems to prove," Schwann remarks, "that the walls of the cells have the power, not merely of chemically changing, but also of sepa-

* De pure et granulatione.—Berol., 1837. † Müller's Archiv, 1839, p. 26.

‡ Froriep's Notiz, Bd. v., p. 225. § Isis, 1838, No. 7.

|| Hufeland's Journal, May, 1838.

rating the matters on which they act, causing some to collect on their interior, and others on their exterior; just as substances separated by the action of galvanism collect at the opposite poles of the battery." (p. 237.)

Such seem to be the principal organic properties manifested by the cells during their growth and subsequent existence within the organism. Now, when it is considered that the entire organism is composed of elements thus endowed, some other important consequences following from M. Schwann's discovery will be readily apprehended. The similarity of the elementary parts of vegetables, in their mode of growth, and in their vital properties, some time since led botanists to regard the cells as the true individuals, and the entire plants as aggregates of these individuals, associated in accordance with a definite law; and vegetables then appeared to differ, in a most important respect, from animals, of which each was viewed as a single individual, and not as an aggregate of many. Since, however, M. Schwann's researches have shown the organism, even of the highest animals, to consist of similar elements, all developed in accordance with the same law, and each endowed with an apparently independent power of growth and self-nutrition, the idea naturally suggests itself, that here, also, the organism is an aggregate of parts endowed with independent vitality; and that its growth and subsistence depend not on any power residing in itself as a whole, but on the independent self-nutrition and reciprocal reaction of its component elements. As a fact strongly confirmatory of this view, Schwann adduces the persistence of vitality and of growth, under favorable circumstances, in single cells or elementary parts separated from the rest of the organism:

"The ova of animals are examples of cells thus manifesting an independent power of growth when separated from the organism to which they belonged. In the case of the ovum of the higher animals, it may certainly be objected that, when fecundated, it can no longer be compared with the other cells of the organism; that fecundation involves something more important than a mere external vital condition, something more than mere nutriment; that, in fact, the ovum may by that act become endowed with its peculiar vitality, and consequently that no conclusions drawn from the ovum can be applied to the other cells of the organism. But, in the case of those genera of animals in which only the female sex exists, and in that of the lower plants which propagate by sporules, there is no ground for this objection. Moreover, amongst the lowest tribes of vegetables, a single cell may separate itself from the plant to which it belonged, and continue an independent growth. In this instance, then, we have demonstrative evidence of the independent vitality of the elementary cells of plants. Now, since all organic cells grow in accordance with the same laws, and consequently cannot owe their growth in one instance to a power of the whole organism, and in another instance to a property resident in themselves; and since, moreover, some of these cells have evidently an independent power of growth, it necessarily follows that we must ascribe to the cells generally the same independent vitality; in other words, we must admit that the particular combination of molecules existing in a cell is sufficient for the development of the power which enables the cell to attract new molecules. Nutrition and growth depend not on a power residing in the organism as a whole, but on the endowments of the individual cells. The circumstance that *every* cell will not continue to grow, when separated from the organism, cannot be urged as an argument against this theory with any more reason than the vitality of a bee can be denied, because it is unable to live separate from the swarm to which it belongs. It proves only that the manifestation of the force inherent in the cell is dependent on certain conditions, which it finds only in its connexion with the entire organism." (p. 228.)

The theory of the independent vitality of the component elements of the organism calls to mind Mayer's hypothesis, that organized bodies are composed of "monads," or "biospheres," which in their active state appear as the globules of the circulating fluids, and in other conditions form the more fixed organic structures. Not merely, however, was Mayer's hypothesis based on few and, in part, erroneous observations, but the absurdity of its details bears testimony rather to the lively imagination than to the soundness of the reasoning powers of its author. Besides ascribing to the individual monads of the organism an independent vitality, it endows them with instinctive impulses, a will, reason, and "a knowledge of the laws of chemistry and mechanics which strikes the mind with astonishment!"

M. Schwann's theory is of an entirely different character. The result of careful induction from numerous and well-ascertained facts, it goes not a step further than those facts and that reasoning warrant. M. Schwann attributes to his cells those properties which constitute what is termed organic life, but not those higher functions which are possessed only by the entire animal organism.

It will have been perceived that M. Schwann rejects the hypothesis of a vital principle, the *anima* of Stahl, by which the various nutritive processes of each animal and vegetable organism have been supposed to be regulated in accordance with rational laws, and that he adopts the opposite view, which seems to be rapidly gaining ground amongst physiologists, namely, that the forces on which the phenomena of life depend obey blindly the laws of necessity, and are, like the forces of inorganic nature, dependent on the existence of matter, though manifested only by a peculiar combination of elements. The adaptation displayed in the processes of organic bodies, M. Schwann remarks, may, like that so remarkable in the planetary system, be due to properties given to matter at its creation; in virtue of which, though obeying blindly the laws of necessity, it concurs in the production of a wisely-ordered whole (p. 222). In addition to other arguments in favour of this view, M. Schwann urges the difficulty of reconciling the hypothesis of a ruling vital principle with the independent vitality and power of growth of the elementary cells of the organism.

Having, as we have already shown, arrived at the conclusion that the force on which the vital or organic phenomena depend resides not in the entire organism, but in its elementary parts or cells, Schwann enters, lastly, upon an enquiry into the nature of that force. Here we shall not follow him, but shall content ourselves with stating the conclusion at which he arrives, namely, that the process by which the cells composing organic beings are formed may be compared to crystallization, and that those organized beings themselves may be regarded as the forms in which matters capable of imbibition crystallize. We should, however, be doing M. Schwann an injustice, if we did not state that he himself views this hypothesis as containing very much that is uncertain and paradoxical, and puts it forth only as a guide to further researches (p. 257).

The length and tenor of the foregoing review of M. Schwann's work evince sufficiently our high estimation of its merits. We shall, therefore, not be suspected of a desire to detract from the high honours due to its author, when we direct attention to a theory of the development of the tissues contained in the writings of Heusinger, which at first view appears like an entire anticipation of the discoveries we have been considering. The following is an extract from the first part of Heusinger's "System der Histologie," published in 1822:

"Matter is the result of the preponderance of the force of contraction over that of expansion. . . . . The equilibrium of these two forces is expressed in the globular form. . . . Hence all organisms and all the elementary parts of organic bodies have at first the globular form. The contest, however, which is expressed in the forces, displays itself also in the matter; hence increased tension of the forces causes the globule (often only apparently homogeneous) to assume the form of a vesicle: and hence all organisms in their development pass from the globular to the vesicular structure. But, whenever in the organism globules and the structureless mass exist, they are led by chemical laws to arrange themselves in a linear order, so as to form fibres. Canals or vessels are produced when vesicles dispose themselves in a similar manner. In accordance with these principles, I refer the tissues of the bodies to three chief formations: 1, that of the structureless matter; 2, that of globules, which includes two groups, the true globules and the fibres; and 3, that of vesicles, in which likewise there are two groups, namely, the true vesicles and the vessels."

On following Heusinger further in his definition of the different groups of organic structures thus marked out, we find other instances of remarkable coincidence between his views and those of Schwann. Thus, his "structureless matter," or "formative tissue," corresponds almost entirely with Schwann's "cytoblastema;" and he describes the process of the formation of vessels in the following terms: "Vessels are vesicles which have arranged themselves in regular series, and have opened into each other. In the formative tissue drops of lymph or globules arise, surround themselves with a polarizing membrane, and become vesicles. These vesicles attract each other, and then their cavities coalesce." . . . .

In the application of his theory to almost all the other tissues, however, Heusinger betrays his ignorance not only of their mode of growth, but also of their structure in the perfect state. In fact, neither he nor any writer at that time was acquainted with the facts which form the basis of M. Schwann's theory. The principle of Heusinger's hypothesis was evidently derived from the speculations of philosophers respecting the forces of inorganic nature, and the statements by which he illustrated its application to organic bodies were founded on imperfect observations, or, in many instances, perhaps, on fancy alone. An hypothesis so ill supported deservedly attracted but little attention, and contributed in no way to the advancement of science. The fate of Schwann's theory has been very different. Deduced by cautious reasoning from well-ascertained facts, and verified by extended investigations, it has already been recognized as the accurate expression of an important law of nature; has found a more extensive application than its discoverer perhaps anticipated; and promises, when followed into its remote consequences, to throw light on many phenomena at present involved in obscurity.

Brit. & For. Med. Review, No XVIII. April 1840.

Printed by C. Hullmandel

21
22
24
25
26
23
27
30
28
31
29
34
32
33
35
36
37
40
39
44
43
41
47
38
46
42
45

### *Explanation of the Figures.*

### Plate II.

Fig. 1. First stage of the formation of cytoblasts from organizable mucus. (Schleiden.)

2. A cytoblast with the cell forming on it. (Schleiden.)

3. A young cell with its cytoblast from *pimelia drupacea.* The cytoblast presents three nucleoli. (Schleiden.)

4. Cell with cytoblast more advanced. (Schleiden.)

5. Progress of organization of albumen in the embryo-sac of *chamædorea schiedeana ; a,* amorphous mass, with granules and cytoblasts beginning to appear, as in fig. 1 ; *b,* new cells still soluble in distilled water ; *c, e,* further progress of the same ; in the last, the cells are flattened against each other. (Schleiden.)

6. Portion of articulated hair from *potato,* showing the course of the currents in the cells. (Schleiden.)

7. Single cell from beaded hair of *tradescantia virginica,* showing the cytoblast (nucleus) and course of the currents. (Slack.)

8. Section of cells in advanced state, showing the cytoblasts imbedded in their walls. (Schleiden.)

9. Portion of ovule of *echium vulgare ; a,* embryo-sac ; *b,* embryo. (Schleiden.)

10. Pollen-tube of *orchis morio,* with cells forming in its interior. (Schleiden.)

11. Development of cells in ovule of *phormium tenax ; a,* embryo-sac ; *b,* pollen-tube ; *c,* embryo. (Schleiden.)

12. Vegetation of yeast in various stages ; *a,* isolated vesicles ; *b,* the same with buds ; *c,* further development of the same. (Turpin.)

13. Cells from leaf of *pinus sylvestris,* showing concentric layers of subsequent deposit. (Meyer.)

14. Cells from stone of plum, with sclerogenous deposit in their interior. (Lindley.)

15. Cells from albumen, showing canals of partial communication. (Schleiden).

16. Cells from gritty matter of pear, showing the radiated form of the cavity. (Lindley.)

17. Duct, showing remains of spiral structure in its walls. (Slack.)

18. Plan of the formation of the dotted duct. (Lindley.)

19. Vessels of the latex, from stipule of *ficus elastica.* (Schultz.)

20. Formation of the same from cells. (Slack.)

### Plate III.

*All the following Figures are copied from Schwann's Plates.*

Fig. 21. Cells of the chorda dorsalis of *cyprinus erythrophthalmus ; a, a,* the nuclei ; *b, b, b,* and *c,* vesicles within the cells, perhaps young cells ; *d,* a small corpuscule contained in the wall of a vesicle.

22. Cartilage of the branchial ray of *cyprinus erythrophthalmus,* taken from near the free extremity of the ray ; *a, a,* nuclei.

23. The same taken from the middle of the branchial ray ; *a,* cavity of a cell ; *b,* its wall ; *c,* a space filled with intercellular substance.

24. The same from the base of the ray ; *a,* walls of the cells.

25. A lamina cut from near the apex of a branchial cartilage of *rana*

*esculenta;* *a*, the border corresponding to the free surface of the cartilage; *b*, a nucleus; *c*, *c*, young cells surrounding nuclei; *d*, granular matter deposited around the nucleus within a cell; *e*, a nucleus apparently in the act of formation around a nucleolus.

26. Nuclei not yet surrounded with cells from the medullary part of the shaft of a growing feather of a raven.

27. Cells from the same part in different stages of development.

28. Cells from the interior of the shaft of a perfectly-developed wing-feather.

29. Cells of the crystalline lens of a fetal pig; *a*, *a*, cells each containing a nucleus; *b*, a nucleus with a young cell developing upon it.

30. A nucleus with two nucleoli, upon which no cell is yet developed, from the crystalline lens of a fetal pig.

31. Cells in the process of transformation into fibres of the lens.

32. Enamel fibres from the imperfectly-formed tooth of a fetal pig.

33. Cells from the surface of the enamel membrane.

34. Fibres which compose the ivory of the human tooth in its early condition, separated by maceration during two days in dilute muriatic acid.

35. Cells undergoing transformation into fibres of cellular tissue, taken from beneath the cutaneous muscle of the neck of a fetal pig.

36. A further stage in the development of cellular tissue, by the elongation and splitting of fibres.

37. Cells apparently undergoing transformation into fibres of elastic tissue, from the middle coat of the aorta of a fetal pig.

38. Flattened cells becoming resolved into fibres, from the cortical substance of the shaft of a young raven's feather; *a*, one of the flattened nucleated cells; *b*, the same in greater part changed into fibres, the nucleus remaining; *c*, the same, absorption of the nucleus commencing; *d*, the same, the nucleus wholly absorbed.

39. Fibres from the dorsal muscles of a fetal pig.

40. The fibre *b* of the preceding figure, after it had been acted on by acetic acid.

41. Muscular fibres further advanced in development, from the brachial muscles of a fetal pig. The fibre *b*, viewed sideways, shows that the nuclei are attached to its walls, and not contained in its cavity. The fibres *a* and *c* illustrate the gradual deposition, on the inner surface of the tube, of the new matter which afterwards forms the ultimate fibrillæ; this deposition is further advanced in fibre *c*.

42. Fasciculus of nervous fibres from the brachial plexus of a fetal pig; *a*, a fibre partially separated from the fasciculus.

43. Single nervous fibres in different stages of development; *a*, from the nervus trigeminus; *b*, *c*, from the nervus ischiadicus of the same fœtus.

44. Nervous fibre from the vagus of a calf, showing nuclei apparently inclosed in its sheath.

45. Pigment cells from the tail of a tadpole, in different stages of development.

46. Ramified pigment cells from the tail of a tadpole; *a*, the point where two cells have apparently coalesced.

47. Diagram representing the mode of formation of capillaries in the area pellucida of a hen's egg; described in the text.

## Figures and Tables

**Figure f1:**
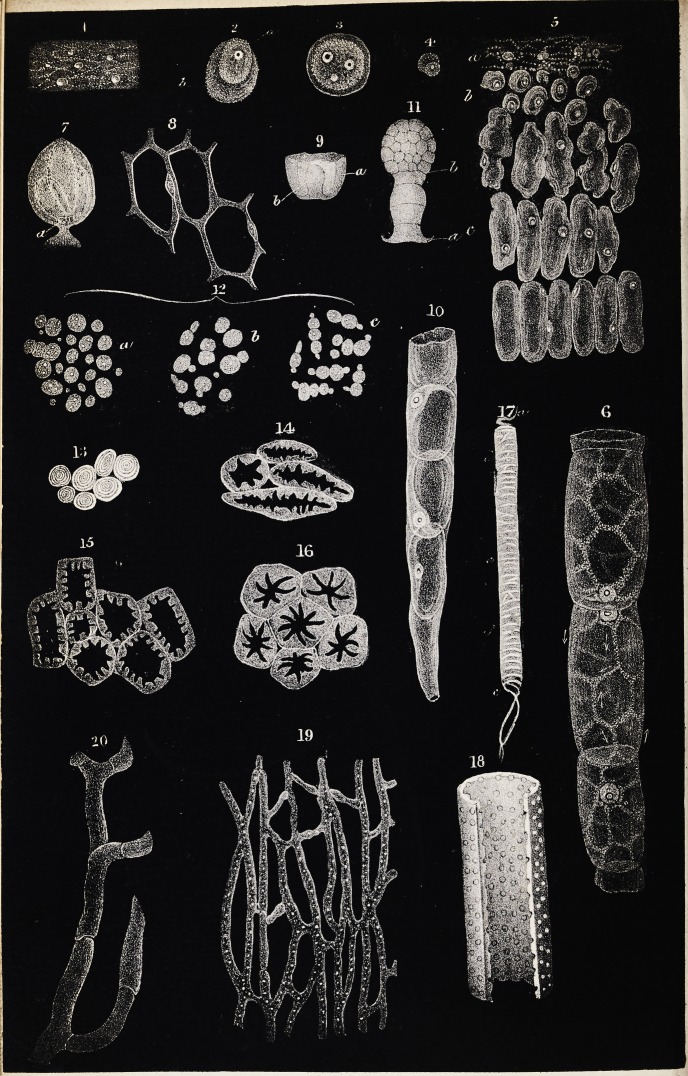


**Figure f2:**